# Supplementary material for: Perceived usability of a store and forward telehealth platform for diagnosis and management of oral mucosal lesions: A cross-sectional study
Source: PLoS One. 2020 Jun 5;15(6):e0233572. doi: 10.1371/journal.pone.0233572 (PMC7274404; doi:10.1371/journal.pone.0233572)
Supplement: S1 Fig — (PDF) [file pone.0233572.s001.pdf]

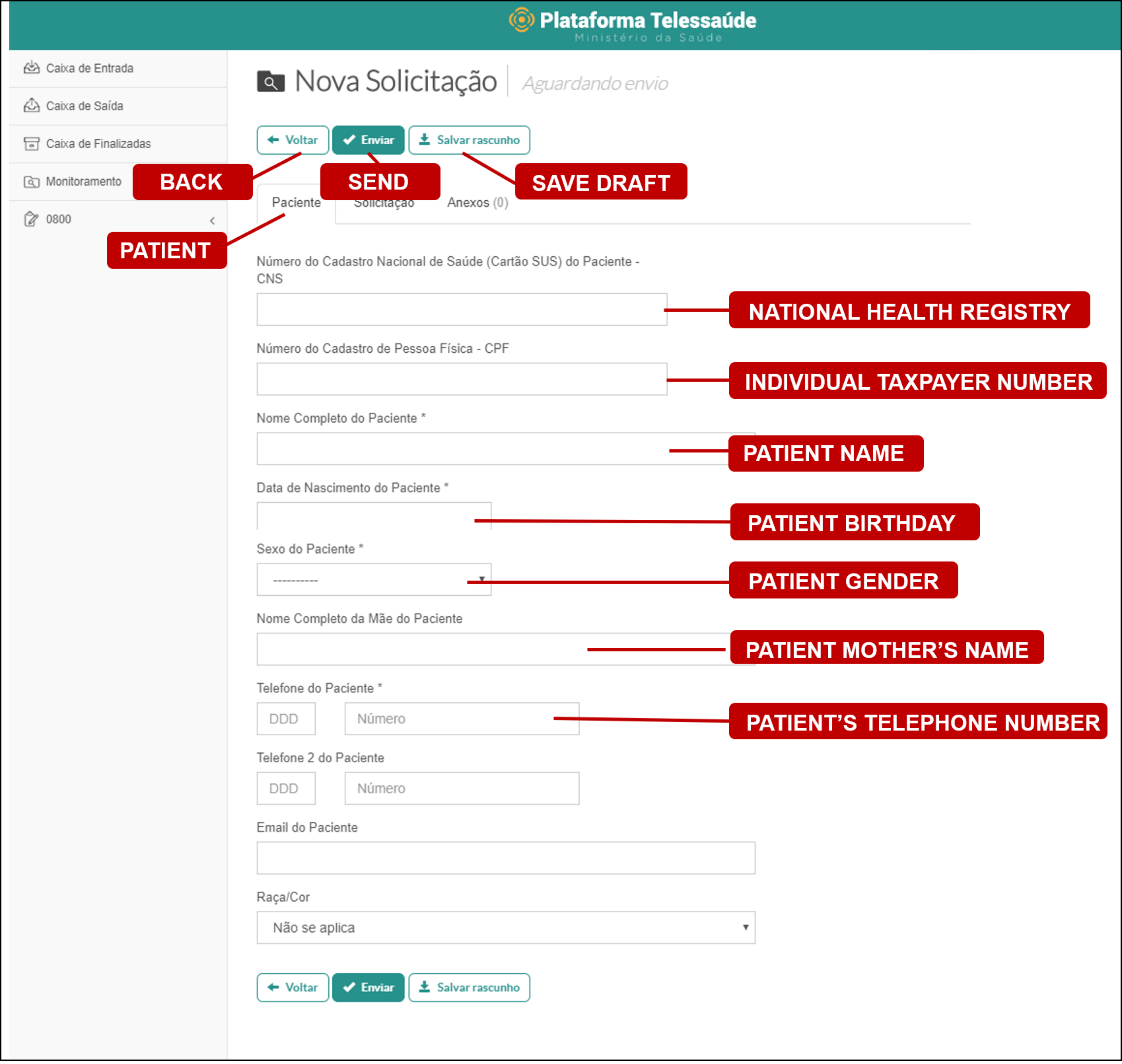
Plataforma Telessaúde
Ministério da Saúde
Caixa de Entrada
Caixa de Saída
Caixa de Finalizadas
Monitoramento
0800
Nova Solicitação
Aguardando envio
Voltar
Enviar
Salvar rascunho
BACK
SEND
SAVE DRAFT
Paciente
Solicitação
Anexos (0)
PATIENT
Número do Cadastro Nacional de Saúde (Cartão SUS) do Paciente - CNS
NATIONAL HEALTH REGISTRY
Número do Cadastro de Pessoa Física - CPF
INDIVIDUAL TAXPAYER NUMBER
Nome Completo do Paciente *
PATIENT NAME
Data de Nascimento do Paciente *
PATIENT BIRTHDAY
Sexo do Paciente *
PATIENT GENDER
Nome Completo da Mãe do Paciente
PATIENT MOTHER'S NAME
Telefone do Paciente *
DDD
Número
PATIENT'S TELEPHONE NUMBER
Telefone 2 do Paciente
DDD
Número
Email do Paciente
Raça/Cor
Não se aplica
Voltar
Enviar
Salvar rascunho

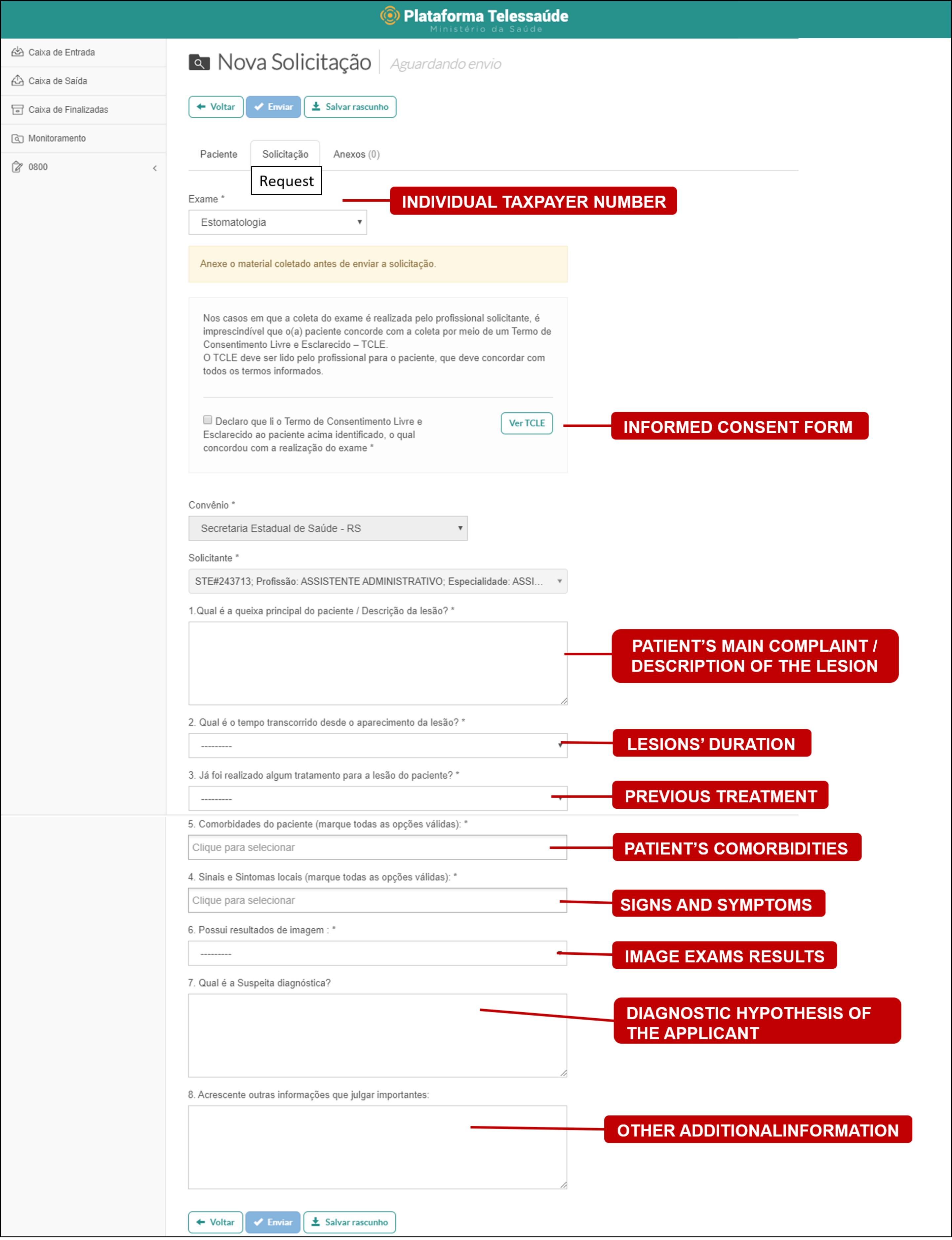
Plataforma Telessaúde
Ministério da Saúde
Caixa de Entrada
Caixa de Saída
Caixa de Finalizadas
Monitoramento
0800
Nova Solicitação
Aguardando envio
Voltar
Enviar
Salvar rascunho
Paciente
Solicitação
Anexos (0)
Request
Exame *
INDIVIDUAL TAXPAYER NUMBER
Estomatologia
Anexe o material coletado antes de enviar a solicitação.
Nos casos em que a coleta do exame é realizada pelo profissional solicitante, é imprescindível que o(a) paciente concorde com a coleta por meio de um Termo de Consentimento Livre e Esclarecido – TCLE.
O TCLE deve ser lido pelo profissional para o paciente, que deve concordar com todos os termos informados.
Declaro que li o Termo de Consentimento Livre e Esclarecido ao paciente acima identificado, o qual concordou com a realização do exame *
Ver TCLE
INFORMED CONSENT FORM
Convênio *
Secretaria Estadual de Saúde - RS
Solicitante *
STE#243713; Profissão: ASSISTENTE ADMINISTRATIVO; Especialidade: ASSI...
1.Qual é a queixa principal do paciente / Descrição da lesão? *
PATIENT'S MAIN COMPLAINT / DESCRIPTION OF THE LESION
2. Qual é o tempo transcorrido desde o aparecimento da lesão? *

LESIONS' DURATION
3. Já foi realizado algum tratamento para a lesão do paciente? *

PREVIOUS TREATMENT
5. Comorbidades do paciente (marque todas as opções válidas): *
Clique para selecionar
PATIENT'S COMORBIDITIES
4. Sinais e Sintomas locais (marque todas as opções válidas): *
Clique para selecionar
SIGNS AND SYMPTOMS
6. Possui resultados de imagem : *

IMAGE EXAMS RESULTS
7. Qual é a Suspeita diagnóstica?
DIAGNOSTIC HYPOTHESIS OF THE APPLICANT
8. Acrescente outras informações que julgar importantes:
OTHER ADDITIONALINFORMATION
Voltar
Enviar
Salvar rascunho
