# Supplementary material for: Perceived usability of a store and forward telehealth platform for diagnosis and management of oral mucosal lesions: A cross-sectional study
Source: PLoS One. 2020 Jun 5;15(6):e0233572. doi: 10.1371/journal.pone.0233572 (PMC7274404; doi:10.1371/journal.pone.0233572)
Supplement: Attachment — Submitted filename: Response to Reviewers.docx [file pone.0233572.s002.docx]

**April 10, 2020**

**Dear Dr. Simone Borsci, Ph.D.**

Academic Editor

PLOS ONE

**Ref: PONE-D-20-04286**

**Title: Usability of a store and forward telehealth platform for diagnosis and management of oral mucosal lesions: a cross-sectional study**

Dear editor,

Please find, attached, the revised version of the manuscript entitled **Usability of a store and forward telehealth platform for diagnosis and management of oral mucosal lesions: a cross-sectional study (**PONE-D-20-04286**).** We would like to thank the comments, which have helped to improve our manuscript. Below, we present our point-by-point response and changes made in order to address the pointed concerns. All alterations are highlighted in the revised version of manuscript.

Thank you for giving us the opportunity to address the concerns from the reviewer. We hope that the revised manuscript will meet the high standards of Plos One. We greatly appreciate your time in reconsidering our manuscript for publication.

We look forward to hearing from you at your earliest convenience.

With kind regards,

Michelle Roxo-Gonçalves

*Teleconsultant in dentistry*

*TelessaúdeRS-UFRGS Program*

*Federal University of Rio Grande do Sul, Brazil*

**Journal Requirements:**

1. *“Please ensure that your manuscript meets PLOS ONE's style requirements, including those for file naming. The PLOS ONE style templates can be found at http://www.plosone.org/attachments/PLOSOne_formatting_sample_main_body.pdf and* [*http://www.plosone.org/attachments/PLOSOne_formatting_sample_title_authors_affiliations.pd*](http://www.plosone.org/attachments/PLOSOne_formatting_sample_title_authors_affiliations.pdf)*”*

**Answer:**

The changes were made according to the author's guideline

2. *“We note that you have included the phrase “data not shown” in your manuscript. Unfortunately, this does not meet our data sharing requirements. PLOS does not permit references to inaccessible data. We require that authors provide all relevant data within the paper, Supporting Information files, or in an acceptable, public repository. Please add a citation to support this phrase or upload the data that corresponds with these findings to a stable repository (such as Figshare or Dryad) and provide and URLs, DOIs, or accession numbers that may be used to access these data. Or, if the data are not a core part of the research being presented in your study, we ask that you remove the phrase that refers to these data.”*

**Answer**

Tables 3, 4 and 5 were added to provide detailed data accordingly.

3. *“Please include captions for your Supporting Information files at the end of your manuscript, and update any in-text citations to match accordingly. Please see our Supporting Information guidelines for more information:* [*http://journals.plos.org/plosone/s/supporting-information*](http://journals.plos.org/plosone/s/supporting-information)*.”*

**Answer:**

We decided to included Tables to address this question, as mentioned above.

Reviewers' comments:

Reviewer #1:

1. *The sample size calculation is based on a referenced manuscript that concluded 80% of a products usability problems can be detected with 4 or 5 subjects. It is not a true sample size calculation and further does depend on the likelihood of problem detection, the latter not estimated in this manuscript.*

**Answer:**

We follow the recommendation of Reviewer 2 regarding sample size in order to address this concern. The related changes are highlighted in yellow in the revised version of the manuscript.

2. *Also regarding the sample size estimation, it is not a sample size to detect either a difference or equivalence in the two outcomes assessed - a comparison between experienced users and inexperienced users in (1) SyUS scores and (2) time to complete the task. Therefore, there is no basis to assess the chance of an erroneous conclusion being reached based on the statistical tests applied to these outcomes. Given the current sample size rationale, these data would be better described descriptively.*

**Answer:**

Changes were made in order to follow the recommendations of both reviewers on this question. Furthermore, the data have now been presented descriptively (mean, SD and Min-Max).

3. *It would appear that the response rate of 33% should read "non-response" rate based on the numbers provided by the authors, the authors should clarify.*

**Answer:**

The recommend changes regarding sampling was performed and the abovementioned parameter was removed of the manuscript’s revised version.

Reviewer #2:

1. *The manuscript proposes a basic but solid assessment of satisfaction in use, which is not the entire assessment of usability, as per ISO standard 9241-11.*

*Overall the article is acceptable however, some raw statements should be removed or better specified.*

*First the title. As authors are not presenting measures of efficiency and effectiveness they may not claim that this study assessed usability but only satisfaction in use or perceived usability.*

**Answer:**

We have changed the title to “Perceived usability of a store and forward telehealth platform for diagnosis and management of oral mucosal lesions: A cross-sectional study”.

2*. I know that the magic number of 5 users to discover 80% of the problems is still going around, but this is an old and complex story, and to make a long story short, NO five users are never enough - at max it could be a starting point. see for instance:*

*- Borsci, S., Macredie, R. D., Barnett, J., Martin, J., Kuljis, J., & Young, T. (2013). Reviewing and extending the five-user assumption: a grounded procedure for interaction evaluation. ACM Transactions on Computer-Human Interaction (TOCHI), 20(5), 1-23.*

*- Schmettow, M. (2012). Sample size in usability studies. Communications of the ACM, 55(4), 64-70.*

*I strongly suggest authors to remove references to the 5 users assumption.*

**Answer:**

We would like to thank for the recommendation which will improve our manuscript. The mentioned references have been removed. Moreover, we performed the calculation proposed by Borsci et al. (2013) considering the findings obtained by the "think out load" evaluation. The four points of difficulty addressed by the participants were used for calculation, as referred by the suggested tool (Fig 5 - Borsci et al., 2013). The related changes are highlighted in yellow in the revised version of the manuscript.
